# Discovery of 16-Androstenes (Androstenone and Androstenol), Their Synthesis Pathway, and Possible Role in Reproduction of Mouse Deer (*Moschiola indica*)

**DOI:** 10.3390/cells11233837

**Published:** 2022-11-29

**Authors:** Vinod Kumar, Shivakumara Manu, Karunakaran Caroline, Anupama Sekhar, Sajwan-Khatri Mamta, Mushkam Sandeep, Wasimuddin, Balasubramanian Senthilkumaran, Govindhaswamy Umapathy

**Affiliations:** 1 Laboratory for the Conservation of Endangered Species (LaCONES), CSIR—Centre for Cellular and Molecular Biology (CCMB), Hyderabad 500007, India; 2 Department of Animal Biology, School of Life Sciences, University of Hyderabad, Hyderabad 500046, India; 3 Nehru Zoological Park, Hyderabad 500064, India

**Keywords:** androstenone, mouse deer, metabolic pathway, reproduction, hormone

## Abstract

We discovered odorous 16-androstenes (Androstenone and Androstenol) in endangered mouse deer during a captive breeding program. This study examined the molecular characteristics, their synthesis pathway, and the possible functional role of these compounds in the reproduction of mouse deer. CYP17A1 and CYB5 genes were cloned and expressed in HEK-293, COS-7 cell lines, and gonads of mouse deer to investigate the CYP17A1 gene’s andien-β-synthase activity towards the synthesis of 16-androstenes in mouse deer. An enzyme immunoassay was further developed and standardized to measure fecal androstenone during the reproductive cycles of mouse deer. Results showed that the mouse deer CYP17A1 gene possesses andien-β-synthase activity and could transform pregnenolone into 5,16-androstadien-3β-ol. The expression of the CYP17A1 gene upregulated in the testis and ovary compared to other tissues in mouse deer. Significantly elevated androstenone and estrogens were recorded prior to delivery and postpartum estrus/mating in mouse deer. Further, there were weak correlations between fecal androstenone and estrogens/androgens in mouse deer during the breeding season. These findings suggest that androstenone probably plays a role in the reproductive activities of mouse deer. This knowledge can be used for captive breeding programs of mouse deer in India and elsewhere.

## 1. Introduction

Pheromones are chemical messengers secreted by an individual to modulate the behavior and physiology of conspecifics [1]. In mammals, pheromones play various important roles in distantly related species, such as in social interactions and organization [2], sexual attraction [3], communication between mother and offspring [4], dominance and aggregation behavior [5], and scent marking and territorial behavior [6]. Pheromones are known to influence the secretion of gonadotropin hormones, testosterone, and luteinizing hormones in mice [7], sea lampreys [8], and sows [9]. A significant relationship between pheromones and reproductive hormones in pigs [10] and Asian elephants [11] was observed. Furthermore, pheromones are known to have a direct influence on various behavioral activities during the estrus cycle in gray opossums [12], Asian elephants [11,13], sows [14], blackbuck [15], mice [16], and bovine [17,18].

Called “boar pheromones”, 16-androstenes are odorous steroids first discovered in pigs that play a major role in social, sexual interactions, and reproduction, [19,20,21,22,23,24]. Subsequently, 16-androstenes were identified in humans with positive effects on females’ mood throughout the menstrual cycle, menstrual synchrony, and male choice performance by activating the hypothalamus in females [25,26,27,28,29,30]. Overall, 16-androstene occurrence plays a possible role in social, psychological, and social impacts on humans [26]. The main components of 16-androstenes (unsaturated C-19 steroids) are 5α-androst-16-en-3-one (Androstenone) and 5α-androst-16-en-3-ol (Androstenol). Additionally, 16-androstenes, particularly androstenone, possess a urine-like smell [22,23] released by the subaxillary salivary gland in large amounts and act as a signaling pheromone to stimulate the estrus in female pigs [19,20,24]. These pheromones are known to have both priming and signaling activity to exhibit potent sexual stimuli in minipig boars (24). It is observed that 16-androstenes accelerate puberty in female pigs, known as the “boar effect” [31,32]. The other pheromone, androstenol, produces a musk-like odor and contributes to the biological effects similar to androstenone in sows [14] and humans [33].

Four key candidate genes are involved in the production of 16-androstenes in pigs, including Cytochrome P450C17 (CYP17A1), Cytochrome B5A (CYB5A), 3β hydroxy dehydrogenase (3β-HSD), and 5α reductases (SRD5A) [34]. The first step in the biosynthesis of 16-androstenes from pregnenolone is catalyzed by the andien-β-synthase enzyme, which, in particular, converts pregnenolone to 5,16-androstadien-3β-ol [35]. The andien-β synthase is the intermediary enzyme between pregnenolone and 5,16-androstadien-3β-ol, which essentially comprises CYP17A1, CYB5A, and their associated reductases: NADPH cytochrome P450 reductase (POR) and NADH cytochrome B5 reductase (CYB5R3) [36,37]. Cytochrome P450c17 possesses two distinct activities: first, the 17α-hydroxylase activity converts pregnenolone to 17α hydroxy pregnenolone and further leads to the synthesis of glucocorticoids while the second activity, the C17-20 lyase activity, breaks the C17-20 bond of C21 steroids by converting 17α-hydroxy pregnenolone to dehydroepiandrosterone (DHEA), the precursor of sex steroids [38]. 

However, despite these dual activities, CYP17A1 possesses a third specific andien-β-synthase activity that catalyzes the transformation of pregnenolone into 5,16-androstadien-3β-ol without any intermediate precursors in humans and pigs [39]. CYP17A1 independently shows poor catalysis (<2%), but it is drastically enhanced by the addition of exogenous CYB5A and POR, revealing a 15% increase in pigs and 12% in humans [39]. Previous studies have shown that the expression of CYP17A1 in pigs is found to be tissue-specific and restricted to gonads (ovary and testis) and adrenal tissues. However, CYP17A1 mRNA expression is equally distributed in the ovary, testis, and adrenal tissues of pigs [40]. 

Mouse deer (*Moschiola indica*), a primitive deer, belong to a distinct family, Tragulidae, listed in Schedule I of the Wildlife Protection act 1972, India and face severe threats due to habitat loss and hunting. As part of the conservation breeding and species recovery program, the Laboratory for Conservation of Endangered Species, CSIR-CCMB, Hyderabad is involved with Nehru Zoological Park, Hyderabad to breed them in captivity. With a founder population of eight individuals, the breeding center has bred more than 400 individuals, of which, more than 100 individuals were released into the wild. We studied the reproductive characteristics of mouse deer as part of the captive breeding program [41] and discovered the occurrence of postpartum estrus within 4–6 h of delivery, which is the shortest observed so far among large mammals. This led us to undertake a study on understanding the reproductive physiology of the mouse deer. While analyzing hormone profiles using gas chromatography-mass spectrophotometry (GC-MS), we discovered androstenone and androstanol in mouse deer, which are reported to play a major role in the reproduction of pigs [19,20]. Thus, we hypothesize that these 16-androstenes could be playing a similar role in the reproduction of mouse deer. With this background, the present study posed the following questions: (1) Do 16-androstenes have a similar molecular pathway of synthesis as reported in pigs and humans? (2) Are 16-androstenes involved in reproductive activities as previously reported in other mammals? (3) Are these compounds expressed in specific tissues in males and females? Based on this, the objectives of the present work are: (1) to examine the molecular characteristics of androstenone and androstenol in mouse deer (2) to characterize the molecular pathway of 16-androstenes production in mouse deer and (3) to measure androstenone and reproductive hormones to understand their role in the reproduction of mouse deer.

## 2. Materials and Methods

### 2.1. Study Animal

The experimental group comprised ten adult females and four adult males of mouse deer (*n* = 14) housed at the mouse deer conservation breeding center of Nehru Zoological Park, Hyderabad (Table 1). They were housed in 15 m (length) × 8 m (width) × 4 m (height) enclosures covered with seminatural roofing partially covered with creepers to maintain a natural photoperiod and light. Visibility was maintained between enclosures by iron mesh so that the animals could see other individuals in neighboring cages. Further, these enclosures also had sub-enclosures within them implemented by iron mesh in the corner (3.5 × 4 m) to hold or separate the male or female for collection of samples. These enclosures were enriched with vegetation, including bamboo (*Bambusa vulgaris*), acalypha (*Acalypha indica*), and royal palm (*Roystonea regia*). The animals were fed twice a day with apple (50 g), banana (100 g), carrot (50 g), sweet potato (50 g), soaked grams (50 g), lucerne (100 g), dry grass (500 g), and peepal (500 g) and had free access to clean water as per the Central Zoo Authority of India norms. All behavioral activities, including mating and estrus, were monitored 24 × 7 using CCTV cameras. Females in an advanced stage of pregnancy were housed with adult males to facilitate mating following parturition [41].

### 2.2. Fecal Sample Collection

Fresh fecal pellets were collected in the morning between 9 and 11 am from 14 experimental animals twice a week except for three weeks prior to parturition when samples were collected daily and stored at −20 °C until further analysis. Extreme caution was taken while collecting fecal samples to avoid urine and human sweat contamination. Details of an individual’s age, sex, number of samples, reproductive behavior, mating, and other information were recorded (Table 1). 

### 2.3. Tissue Collection 

Gonad tissues (testes and ovary) and heart tissues were collected opportunistically from Nehru Zoological Park during the study period. The tissues were harvested during postmortem in RNA*later* stabilization solution (Thermo Fisher Scientific, Waltham, MA, USA) on ice and stored at −80 °C until further analysis. For GC-MS analysis, 16-androstenes (androstenone and androstenol) were extracted using the previously modified procedure [42]. Approximately 4 g of frozen tissues in triplicates were homogenized using liquid nitrogen followed by adding 10 mL of methanol and centrifuging at 4000 rpm for 10 min. The supernatant was transferred into a fresh glass tube, then 3 mL of diethyl ether was added and recentrifuged to collect the supernatants (Ether layer). The procedure was repeated at least two times. All supernatants were collected in one tube and dried under the stream of nitrogen and resuspended in 20 µL of methanol and used in GC-MS.

### 2.4. Extraction of Androstenone and Steroid Metabolites

Fecal hormone metabolites were extracted using the previously described procedure [43]. The pulverized fine fecal powder was weighed at approximately 0.2 g in a 15 mL glass tube, then 3 mL of 80% methanol was added to it. The samples were vortexed for 30 min at room temperature and kept overnight at 4 °C. The next day, the samples were centrifuged at 3300× *g* for 20 min, transferred to a fresh tube, and kept at −20 °C until further analysis. 

### 2.5. Identification of 16-Androstenes Using GC-MS

The 16-androstenes (androstenone and androstenol) were identified using the GC-MS system (Agilent 6900 Agilent Technologies, Santa Clara, CA, USA). Before injecting into GC-MS, approximately 10 mL of pooled fecal extracts were purified by solid-phase extraction using a Sep-Pak C18 matrix column (Waters, Milford, MA, USA). In brief, fecal extracts were passed through the C18 column at 1 mL/5 min flow rate (per manufacturer’s instructions) and eluted with 5 mL of 90% methanol in order to remove the contaminants. The purified supernatant samples were then evaporated under nitrogen gas and resuspended in 100 µL of absolute methanol and kept at −20 °C until further analysis [44]. This method was specifically designed to minimize the loss of volatile 16-androstenes and to recover the maximum quantity of steroids during purification [42]. 

Extracted fecal samples were analyzed using an HP-5 MS capillary column with a 30 m × 250 µm × 0.25 µm film thickness (Agilent Technologies, Santa Clara, CA, USA). Helium was used as a carrier gas with a head pressure of 15.8 psi and injector temperature of 300 °C. The interface and ion source temperature were maintained at 300 °C and 200 °C, respectively. The injection was made, and after 3 min, purge valve was turned on, then 2 µL of each sample extract was injected in the split-less mode with an inlet temperature of 270 °C. The oven temperature was raised to 300 °C at 15 °C/min. The mass spectra were recorded in full scan mode and identified by computer MS library search (Wiley MSD Chem 7th Edition) and authentic standards (Sigma-Aldrich Chemical Co. St. Louis, MO, USA) were used to confirm the desired compounds by comparison of spectra and retention time of samples.

### 2.6. Total RNA Isolation and cDNA Synthesis

Total RNA was extracted from the tissues (testis, ovary, and heart) using the trizol (Invitrogen, Carlsbad, CA, USA) method. RNA concentration and quality were evaluated by an ND-1000 Spectrophotometer (NanoDrop Technologies, Wilmington, DE, USA). After DNase treatment (DNase I amplification grade, Invitrogen), cDNA synthesis was carried out using the first-strand cDNA synthesis kit (Takara Bio, San Jose, CA, USA) from 2 µg of the total RNA and incubated at 42 °C for 1 h followed by 95 °C for 5 min. 

### 2.7. Targetted Assembly of Candidate Gene Exons from Shotgun Sequencing

As no CYP17A1 and CYB5A gene sequences of mouse deer or any closely related species were available in Genbank, shotgun sequencing was performed of mouse deer genomic DNA to assemble the candidate gene exons for designing primers specific to mouse deer. Briefly, genomic DNA was isolated from a tissue sample using DNeasy Blood and Tissue Kit (Qiagen, Hilden, North Rhine-Westphalia, Germany) and quantified using a dsDNA assay in Qubit 4 Fluorometer (ThermoFisher Scientific, Waltham, Massachusetts, USA). About 1µg of genomic DNA was taken as input for library preparation using Truseq DNA PCR-free kit (Illumina, San Diego, CA, USA) following the manufacturer’s protocol. The purified library was quantified using the NGS library quantification kit (Takara Bio, San Jose, California, USA) and pooled with other libraries to obtain about 50× coverage. The pooled libraries were sequenced on an S4 flow cell on the Illumina Novaseq 6000 platform. The first and last exons of CYP17A1 and CYB5A were assembled by taking the corresponding exons from pig reference gene sequences as a template using the selective recursive local assembly of homologous genomic regions (SR Assembler) [45]. The primers were designed using the assembled sequences of the first and last exon to amplify the whole CDS from the first codon to the last codon, excluding the stop codon. Kozak sequences were added to the forward primer to enable transcription after cloning into an expression vector.

### 2.8. Construction of CYP17A1 and CYB5A 

The cDNA fragments possessing the entire coding region of CYP17A1 and CYB5A of mouse deer and pig testis were amplified using LA taq DNA polymerase (Invitrogen) with primers listed in Table 2. The coding sequences of CYP17A1 and CYB5A with the Kozak regions were then amplified and the segments were cloned into expression vectors pcDNA3.1/V5-His TOPO (Invitrogen, Waltham, MA, USA) to produce the expression vectors. 

### 2.9. RT-PCR for Quantifying CYP17A1 Gene Expression in Tissues

To quantify levels of gene expression of CYP17A1 in different tissues of mouse deer and pig, we designed primers in the conserved exon-–exon junctions of mouse deer and pigs. CYP17A1 and β-actin were used as reference genes for control. The primers spanning the exon–exon junction would only amplify cDNA and would not amplify any contaminated genomic DNA. RT-PCR was performed to examine the expression levels of CYP17A1 in testis and ovary in mouse deer using the TB Green Premix Ex Taq II (Takara Bio, San Jose, CA, USA) following the manufacturer’s protocol. Heart tissue was used as internal control. The PCR reaction composed of 5 µL of SYBR Premix Ex Taq, 1 µL of cDNA template, 0.5 µL of reverse primer (10 µM conc.), 0.5 µL of forward primer (10 µM conc.), and 3 µL of milliQ water in a total volume of 10 µL using Roche Lightcycler 480 II (Roche Molecular Diagnostics, Mannheim, Germany). Quantitative PCR conditions were set at 95 °C hot start for 30 s, followed by 40 cycles at 95 °C for 15 s, and 60 °C for 30 s, 95 °C for 5 s, 60 °C for 1 min, and at 50 °C for 30 s. The PCR amplification products were analyzed by cp value and melting curves. 

### 2.10. Functional Characterization of Mouse Deer CYPA17A1 and CYB5A Expressed in Mammalian Cells

HEK-293 and COS-7 cells were grown in DMEM medium supplemented with 10% fetal bovine serum. Cells were transfected using lipofectamine (Promega, Madison, WI, USA) as a transfection agent with pcDNA3.1 (Mock, without gene constructs) and pcDNA3.1 encoding mouse deer CYPA17A1 and CYB5A gene constructs. After 48 h of incubation, fresh medium was added and 50,000 cells/well of 50 µg of ^3^H pregnenolone was added in the fresh media to the culture plates and kept for incubation at 30 °C in 5% Co_2_ incubator for 16 h [46]. After incubation, cells were harvested and separated at 1000× *g* for 5 mins. Further, steroids in the medium were extracted twice with 4 mL of diethyl ether and organic phase was pooled and evaporated to dryness using a stream of nitrogen. The extracts were reconstituted with 100 µL of 85% acetonitrile:15% H_2_O [46]. The separation and identification of steroids were performed using Shimadzu CTO-10AS HPLC-ECD system (Shimadzu Corporation, Tokyo, Japan) on a Luna 5 µm 100 mm × 4.60 mm reverse phase C-18 column (Phenomenex, Torrance, CA, USA). The 16-androstenes were separated from pregnenolone using a mobile phase of 85% acetonitrile at 1 mL/min flow rate (46). The presence of 5,16-androstadien-3β-ol in mammalian cells was further confirmed by GC-MS. Respective standards were used as a reference steroid (Steraloids, Newport, RI, USA) for the identification of 16-androstenes at 200 nm of wavelength.

### 2.11. Androstenone Antibody Production 

Androstenone polyclonal antibody was produced to develop the sensitive indirect competitive enzyme immunoassay (EIA). Two adult New Zealand white rabbits were immunized with 16, (5α)-androsten-3-one-carboxymethyloxime: BSA conjugate (Steraloids, Newport, RI, USA). The first injection was prepared at a concentration of 1 mg/mL of conjugate dissolved in saline and emulsified with an equal volume of Freund’s complete adjuvant (Sigma-Aldrich Chemical Company, St.-Louis, MO, USA). The emulsified solution was injected subcutaneously at four sites on the rabbit’s back. The booster shots (0.5 mg/mL) were administered with Freund’s incomplete adjuvant every two weeks after the initial dose on days 14, 28, 42, 56, and 70. Blood was collected every one week of booster dose on days 35, 49, 63, and 76. [47,48]. The blood was incubated at room temperature for 4–6 h and vortexed at 4000 rpm to isolate the serum. The polyclonal immunoglobin (IgG) antibodies were purified using protein-A affinity chromatography (Pierce, Waltham, MA, USA). The final concentration of purified anti-androstenone antibody was found to be 25 mg/mL, divided into aliquots, and kept at −80 °C. The checkerboard titrations were performed to determine the optimal antibody, conjugated antigen, and secondary antibody dilutions in the androstenone assay. 

### 2.12. Androstenone EIA and Procedure

Fecal androstenone was measured using polyclonal anti-androstenone antibody diluted to 1:25,600, androstenone standards (1000–3.9 ng/mL), 16(5α)-androsten-3-one-carboxymethyloxime: BSA conjugate (1 mg/mL), and horseradish peroxidase (HRP)–conjugated goat antirabbit IgG secondary antibody (geneilabs, bangalore) diluted to 1:10,000. The procedure of 5α-androst-16-en-3-one indirect competitive ELISA was performed as previously described [48]. The 96- well microtiter plate (Nunc Maxisorp; Immuno plate, Waltham, MA, USA) was coated with 100 µL of 1 µg conjugate/mL of 16 (5α)-androsten-3-one-carboxymethyloxime: BSA (diluted in coating buffer, 0.05 M sodium bicarbonate buffer, pH 9.6), placed in a moist chamber and covered with cling wrap, and incubated overnight at 4 °C. The contents of the plate were discarded and washed four times with wash buffer (0.15 M NaCl, 0.05% Tween 20) using an automated ELISA washer (Elx50, BioTek, Winooski, VT, USA). The plate was blocked with 200 µL blocking buffer and incubated at 37 °C for 1 h. The contents of the plate were discarded and the plate was blotted and dried in air. Subsequently, 50 µL of the diluted fecal sample (final dilution 1:4 in EIA buffer) and 50 µL of 5α-androst-16-en-3-one standards followed by 50 µL of diluted 5α-androst-16-en-3-one antibody were added and incubated at 37 °C for 90 min. The plate was again washed with wash buffer, blotted, and 100 µL of HRP conjugated goat anti-rabbit IgG secondary antibody (geneilabs, bangalore) was added to each well and incubated at 37 °C for 90 min. The plate was washed as mentioned above, and 100 µL of substrate solution TMB/H_2_O_2_ (Tetramethylbenzidine/Hydrogen peroxide, geneilabs) was added and incubated in the dark for 5–10 min (for color development). The reaction was stopped using 50 µL of stopping solution (1N HCL) and absorbance was read at 450 nm in the ELISA reader (Thermo Multiskan Spectrum Plate Reader, version 2.4.2, Thermo Scientific, Vantaa, Finland).

### 2.13. EIA for Progesterone, Estradiol, and Testosterone

Fecal progestogens, estrogens, and androgens were measured using the previously described methods [49,50,51,52]. Antibodies and HRP conjugates for progesterone (Monoclonal progesterone, Quidel clone no. 425), estradiol (polyclonal estradiol, R0008), and testosterone (polyclonal testosterone, R156/7) were provided by Dr. Coralie Munro (University of California, Davis, CA, USA). The monoclonal progesterone antibody was diluted to 1:6000 and 1:100,000 for horseradish peroxidase (HRP) conjugated progesterone and standards (200–0.39 pg/well). Cross-reactivity of progesterone antibody was described as previously reported by Graham et al., 2001. The polyclonal estradiol antibody was diluted to 1:10,000 and 1:100,000 for HRP conjugated estradiol and standards (1000–1.95 pg/well). Cross-reactivity of estradiol antibody was with estradiol (100%), estrone 3%, and <1% in others [52]. The testosterone antibody was diluted to 1:10,000 and 1:200,000 for HRP conjugated testosterone and standards (600–1.17 pg/well). Cross-reactivity of testosterone was reported previously [49,50,51]. The procedure for EIAs was performed as previously described [46,47,48].

### 2.14. EIA Validation for Androstenone, Progesterone, Estradiol, and Testosterone

The cross-reactivity of 5α-androst-16-en-3-one antibody with other steroids was assessed using the half displacement method as described earlier [48,53]. The androstenone EIA was validated by demonstrating parallel displacement curves between pooled serial dilution of fecal extracts (endogenous) and respective standard (exogenous) to determine the immunological activity of endogenous antigen with the corresponding antibody used in the assay and fecal sample dilution at 50% binding. Antibody sensitivity was calculated for subjective determination at 90% maximum binding. The accuracy or recovery of the assay was measured by adding known amount of unlabeled steroids to the fecal extract. Details of androstenone antibody cross-reactivity to other C19 and various other steroids have been shown in Table 3. Antibody sensitivity at 90% binding was found to be 3.9 ng/mL, 7.8 pg/mL, 23.4 pg/mL, and 39 pg/mL for androstenone, progesterone, testosterone and estradiol, respectively.

All the EIAs were validated by demonstrating parallelism between pooled standards and serial dilution of fecal extracts (r^2^ = 0.99) (Supplementary Figure S1. Recoveries of known amount of unlabeled steroids were 84.91 ± 4.33, 81.24 ± 2.84, 92.34 ± 5.86, and 98.84 ± 8.51 for androstenone, progesterone, testosterone, and estradiol, respectively, in fecal extracts analyzed by EIA. The correlation (r^2^) and slope (*m*) values for the exogenous androstenone, progesterone, testosterone, and estradiol were r^2^ = 0.99, *m* = 0.86; r^2^ = 0.99, *m* = 0.93; r^2^ = 0.99, *m* = 1.05; r^2^ = 0.98, *m* = 1.15, respectively. The intra- and interassay coefficients of variation (CV) were 6.09% and 10.26% (*n* = 10), 7.64% and 14.63% (*n* = 10), 8.87% and 14.93% (*n* = 10), and 8.385 and 15.89% (*n* = 10) for androstenone, progesterone, testosterone, and estradiol, respectively. The presence of androstenone, progesterone, testosterone, and estradiol in fecal samples was confirmed by HPLC profile and eluted fractions showed the immunoreactivity of fecal hormones with the corresponding antibody (Supplementary Figure S2). 

### 2.15. Statistical Analysis

Transformation activity assay levels were examined using mean ± SEM of three independent experiments. A post-hoc Scheffe test was used for comparing transformation levels of CYP17A1 between control vs. CYP17A1 in mouse deer and control vs. CYP17A1 in pigs in HEK-293 and COS-7 cell lines. Hormone values are presented as mean ± SEM. Weekly mean was calculated in all males and females for hormones. Spearman rank correlation coefficient (rs) test was performed to calculate the correlation between individual females androstenone and estrogens and male androstenone and androgens, respectively. Mann–Whitney U test (M–W test) was used for testing differences in fecal progestogens concentrations in pregnant and nonpregnant animals. Statistical analyses were carried out using SPSS 17.0 for hormone analysis. 

## 3. Results

### 3.1. Identification of 16-Androstenes Using GC-MS

**Figure 1:**
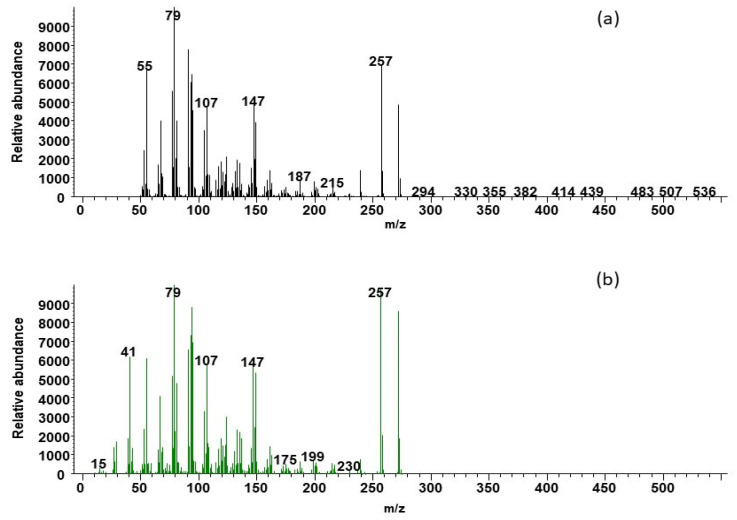
Mass spectra of Androstenone of mouse deer feces (**a**) compared with synthetic androstenone (**b**).

**Figure 2:**
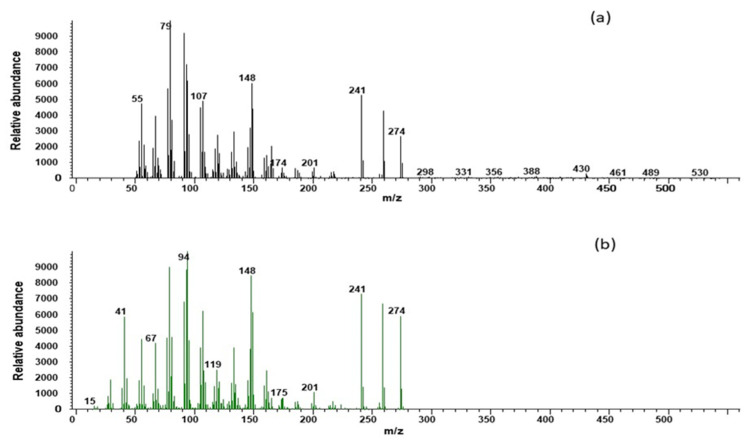
Mass spectra of Androstenol of mouse deer feces (**a**) compared with synthetic androstenol (**b**).

Following GS-MS mass spectra analysis, 16-androstenes (Androstenone and Androstenol) were identified in the feces of mouse deer of both sexes (Figure 1 and Figure 2) as well as androstenone in the tissues of the testis. The androstenone and androstenol were eluted at 4.97 and 4.78 min, respectively (Figure 1a and Figure 2a). The relative retention times of the feces androstenone and androstenol were identical with standards (Figure 1b and Figure 2b). Androstenone and androstenol showed a base peak value at *m/z* 79, *m/z* 257 and *m/z* 79, *m/z* 148, respectively. 

### 3.2. Cloning of CYP17A1 and CYB5A in Mouse Deer 

**Figure 3:**
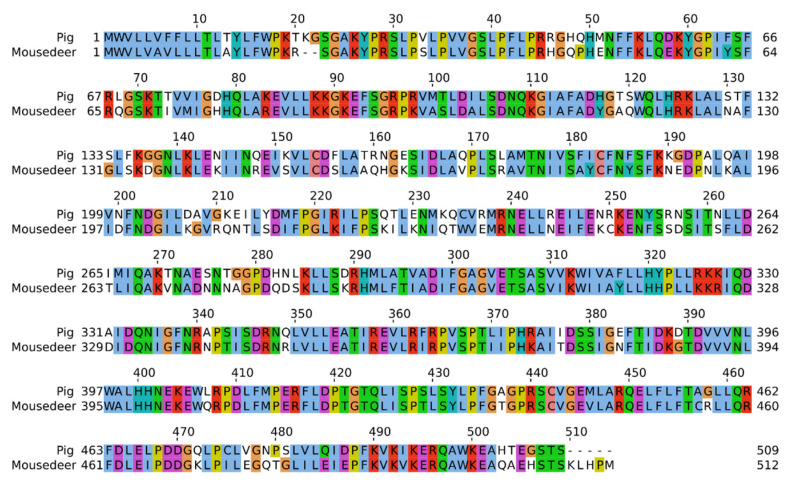
The comparison of deduced amino acid sequence of CYP17A1 between mouse deer and pig. The homologous sequences between mouser deer and pig are highlighted.

**Figure 4:**
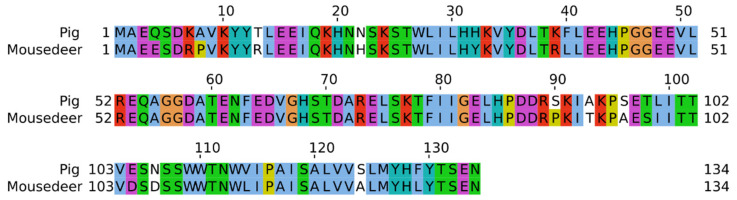
The comparison of deduced amino acid sequence of CYB5 between mouse deer and pig. The homologous sequences between mouser deer and pig are highlighted.

Figure 3 shows the deduced amino acid sequence of mouse deer CYP17A1 compared with pigs using multiple sequence alignment. The full-length transcript amino acid sequence of mouse deer and pig CYP17A1 were found to be 512 (Genbank accession number: ON804800) and 509 amino acids, respectively. The mouse deer CYP17A1 sequence had 73.8% homology with the pig sequence, and it has five extra amino acids (Lys, Leu, His, Pro, Met) as compared to pig. Further, there was a deletion of two amino acids at the 21st and 22nd positions of CYP17A1 in mouse deer with reference to the pig sequence. In addition, a full-length transcript amino acid sequence of CYB5A in mouse deer was found to be 134 amino acids (Genbank accession number: ON804801) (Figure 4), which showed 86.57% sequence homology with pigs. 

### 3.3. Conversion of 5,16-Androstadien-3β-ol by CYP17A1 Mouse Deer, In Vitro

**Figure 5:**
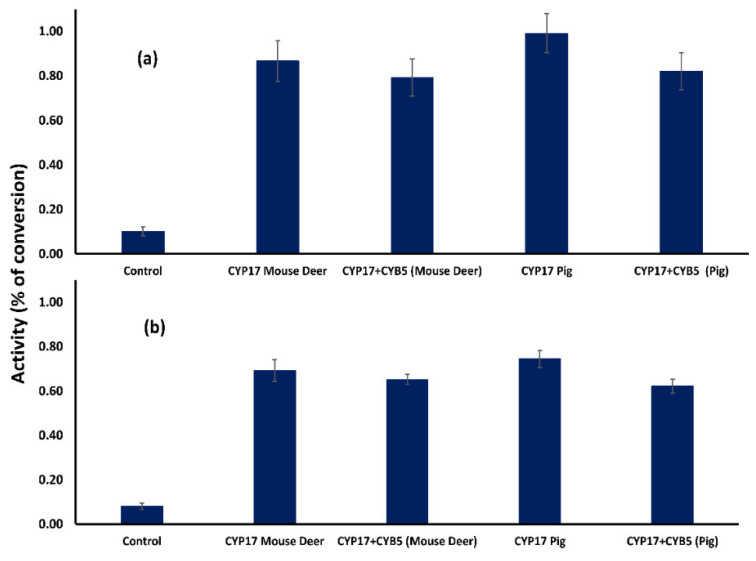
Transformation of 5,16-androstadien-3β-ol from pregnenolone by mouse deer and pig CYP17A1 and effect of CYB5A in combination with CYP17A1 in (**a**) HEK-293 and (**b**) COS-7 cells. The results are the mean ± SEM of three independent experiments. An empty vector was used as a control.

To evaluate the enzymatic activity of CYP17A1 in mouse deer for conversion of pregnenolone into 5,16-androstadien-3β-ol, two mammalian cell lines, HEK-293, and COS-7, were transfected with an expression construct of CYP17A1. In mouse deer, the CYP17A1 enzyme is able to transform pregnenolone into 5,16-androstadien-3β-ol in the presence of cofactors, glucose-6-phosphate salt, glucose-6-phosphate dehydrogenase, and nicotinamide adenine dinucleotide phosphate (NADP) salt (Figure 5). However, the observed transformation was less than 1%. The transformation of 5,16-androstadien-3β-ol from pregnenolone was significantly higher in mouse deer and pig than the control in the HEK-293 cell line (post-hoc Scheffe test; *p* = 0.024, *n* = 5) and COS-7 cell line (post-hoc Scheffe test; *p* = 0.039, *n* = 5). Interestingly, no increase was found in 5,16-androstadien-3β-ol in the presence of CYB5A. Further, no significant difference was observed in the transformation of 5,16-androstadien-3β-ol in HEK-293 and COS-7 cells (M–W U = 14; *p* = 0.9). Overall, results showed that mouse deer P450C17 has a catalytic activity to transform 5,16-androstadien-3β-ol in the absence of oxidoreductase enzymes POR and CYB5R. 

### 3.4. Relative mRNA Transcript Expression of CYP17A1 in Testis and Ovary of Mouse Deer

**Figure 6:**
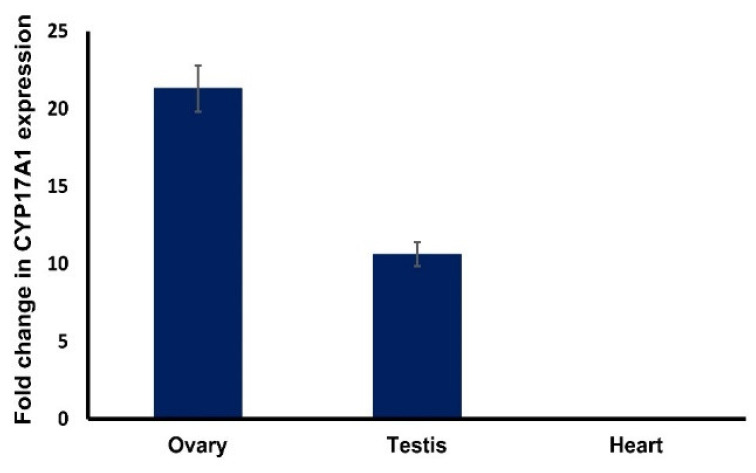
Normalized expression of mouse deer CYP17A1 with respect to β-actin in ovary, testis, and heart.

Mouse deer CYP17A1 mRNA transcript shows tissue-specific expression in the testis and ovary as compared to the heart (as a control) (Figure 6). The expression of CYP17A1 mRNA was restricted to the gonads of mouse deer and did not express in the heart tissue of mouse deer.

### 3.5. Reproductive Monitoring

A total of 1627 fecal pellets were collected from 10 adult females and four adult male mouse deer for 12 months (Table 1). Of the 10 females, eight females were observed with 13 parturitions, postpartum estrus observations, and successful mating (Table 1) during the year of the study period. 

### 3.6. Fecal Androstenone Profile during Parturition, Postpartum Estrus, and Mating

**Figure 7:**
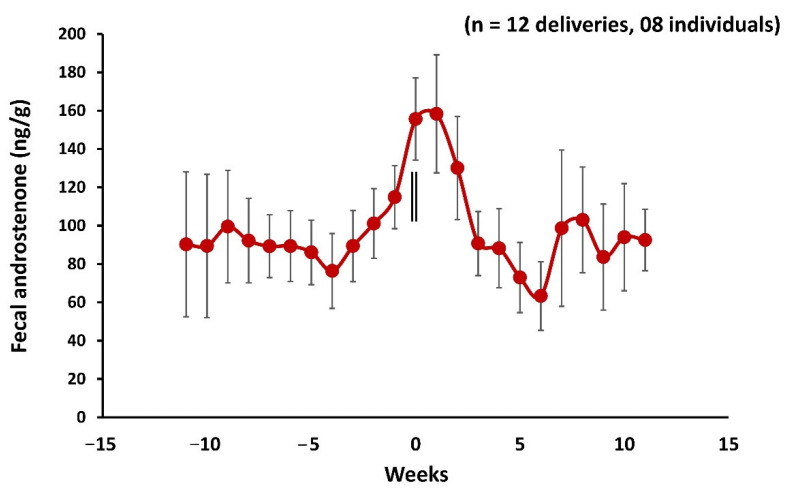
Profile of mean ± SEM weekly fecal androstenone in mouse deer during 11 weeks before and post parturition. The zero week and vertical bars indicate the parturition, mating, and postpartum estrus (*n* = 12) were recorded in eight individuals.

The mean fecal androstenone concentrations ranged between 47.66 ± 4.08 ng/g and 226.11 ± 14.55 ng/g. Figure 7 shows the fecal androstenone concentrations (weekly mean ± standard error of mean (SEM)) of adult female mouse deer from 11 weeks prior to parturition to 11 weeks post parturition. The zero week indicates the week of parturition and mating. A gradual increase of fecal androstenone concentrations began on the third week and led to a sharp increase on day 0 (equivalent of week −3 to day 0 in Figure 7) (3 week vs. 0 week, M–W U = 34, *p* = 0.03, *n* = 8; 2 week vs. 0 week, M–W U = 43, *p* = 0.05, *n* = 8) and significantly decreased on the third week from 0 week (0 week vs. 3 week, M–W U = 30, *p* = 0.03, *n* = 8). In addition, the presence of androstenone was confirmed in the fecal samples and examined androstenone antibody immunoreactivity against the fecal androstenone using high-performance liquid chromatography (HPLC).

### 3.7. Fecal Estrogens during Postpartum Estrus and Mating

**Figure 8:**
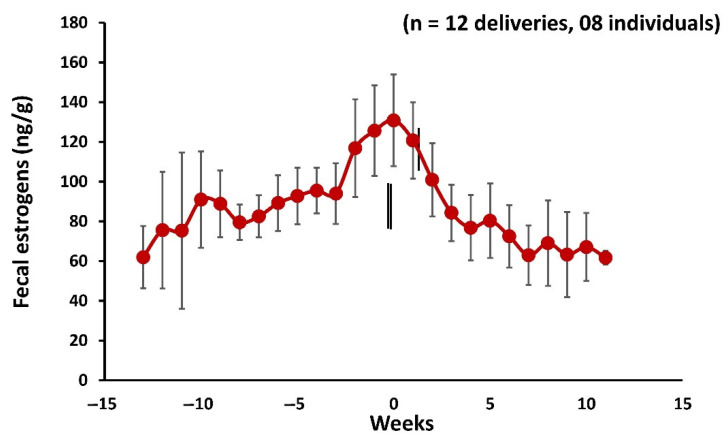
Profile of mean ± SEM weekly fecal estrogens in mouse deer during 11 weeks before and after parturition. The zero week and vertical bars indicate the parturition, mating, and postpartum estrus (*n* = 12) were recorded in eight individuals.

Overall, fecal estradiol metabolite concentration ranged widely from 57.10 ± 2.65 ng/g to 189.91 ± 8.42 ng/g (Figure 8). There was a transient rise of fecal estrogens in females exhibiting estrus. Fecal estrogens levels were elevated for about three weeks prior to parturition, peaked on the day of postpartum estrus, and then declined by three weeks of parturition (−3 vs. −1 week, M–W U = 31, *p* = 0.05, *n*= 8; −3 vs. 0 week, M–W U = 30, *p*= 0.04, *n* = 8; 0 week vs. 3 week, M–W U = 25, *p* = 0.03, *n* = 8). However, all individual females show estrogens peak within 1.33 ± 0.18 (*n* = 12) days following delivery. The peak observed in fecal estrogens levels could be of follicular origin, as mating was observed within 4–6 h of parturition.

### 3.8. Fecal Androstenone vs. Fecal Estrogens

**Figure 9:**
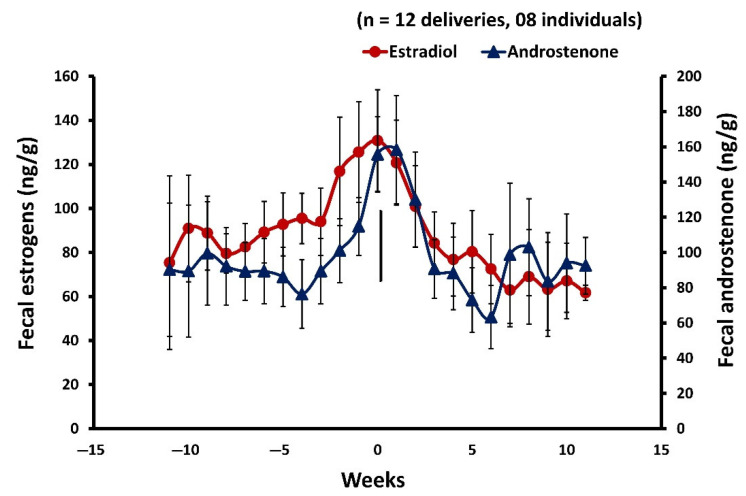
Profile of mean ± SEM weekly fecal androstenone vs. estrogens in mouse deer during 11 weeks before and after parturition. The zero week and vertical bars indicate the parturition, mating, and postpartum estrus (*n* = 12) were recorded in eight individuals.

Overall, 10 out of 13 deliveries from six female individuals showed a weak positive correlation between fecal androstenone and estrogens concentrations one week prior to parturition, postpartum estrus, and mating (Figure 12) (rs = 0.78, *p* = 0.008; rs = 0.24, *p* = 0.489; rs = 0.87, *p* = 0.001; rs = 0.24, *p* = 0.489; rs = 0.18, *p* = 0.603; rs = 0.40, *p* = 0.244; rs = 0.09, *p* = 0.803; rs = 0.30, *p* = 0.385; rs = 0.15, *p* = 0.676; rs = 0.22, *p* = 0.533, *n* = 6 individuals, 10 deliveries, Figure 9)

### 3.9. Fecal Androstenone and Testosterone in Males

**Figure 10:**
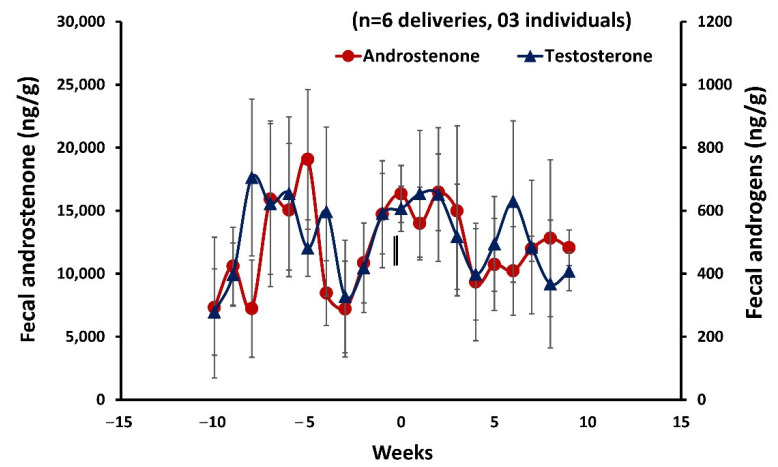
Profile of mean ± SEM weekly fecal androstenone and androgens in male mouse deer during 10 weeks with respect to parturition in females. The zero week and vertical bars indicate the parturition and mating in females.

Individual mean fecal androstenone concentrations ranged from 5560.84 ± 278.44 ng/g to 16,027.87 ± 1109.119 ng/g and began increasing 5-6 weeks prior to mating. Moreover, individual mean fecal androgens concentrations ranged from 311.31 ± 29.85 ng/g to 1069.76 ± 26.32 ng/g (Figure 10). Overall, all individual males showed a weak positive correlation between fecal androstenone and androgens concentrations (rs = 0.90, *p* = 0.002; rs = 0.42, *p* = 0.289; rs = 0.476, *p* = 0.233; rs = 0.762, *p* = 0.028; rs = 0.262, *p* = 0.531; rs = 0.929, *p* = 0.001, *n* = 3 individuals, 6 deliveries)

### 3.10. Fecal Progestogens Profile during Pregnancy and Postpartum Estrus

**Figure 11:**
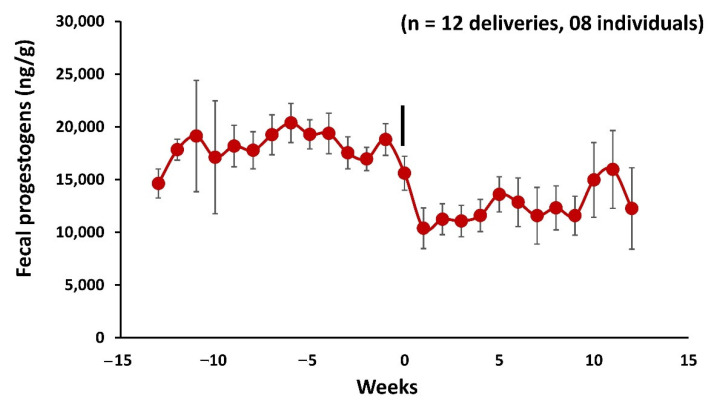
Profile of mean ± SEM weekly fecal progestogens in mouse deer during 11 weeks before and after parturition. The zero week and vertical bars indicate the parturition, mating, and postpartum estrus (*n* = 12) were recorded in eight individuals.

**Figure 12:**
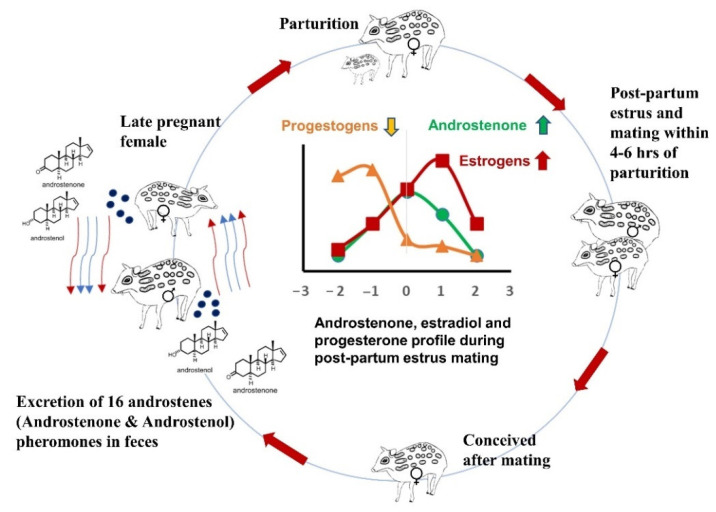
The graphical representation of a model how androstenone (16-androstenes), estrogens, and progestogens play a crucial role during parturition, postpartum estrus, and mating in mouse deer.

Overall, fecal progesterone metabolite concentrations ranged from 4116 ± 204.97 to 20,175 ± 1083.92 ng/g (Figure 11). The mean fecal progesterone metabolite concentrations declined significantly from the day of parturition from 18,067 to 10,207 ng/g (−1 week vs. 1 week, M–W U = 22, *p* = 0.00, *n* = 8, Figure 12). 

## 4. Discussion

Pheromones play a major role in reproduction in mammals [7,9,13]; however, a few studies have demonstrated pheromone synthesis molecular pathways and their specified role in reproduction. In this study, two odorous 16-androstenes (androstenone and androstenol) were identified in mouse deer which showed that the mouse deer P450C17 gene possesses an andien-β-synthase activity to transform pregnenolone to 5,16-androstadien-3β-ol, although the 5,16-androstadien-3β-ol is considered to be an intermediate and precursor in the 16 androstene pathway leading to the biosynthesis of androstenone and androstenol [39]. For the first time, we show that POR and CYB5R are not essential for the conversion of pregnenolone to 5,16-androstadien-3β-ol using P450C17 in mouse deer and pigs. Soucy et al., 2003 [39] showed that pig and human CYP17A1 possesses andien-β-synthase activity that catalyzes the conversion of 5,16-androstadien-3β-ol from pregnenolone. However, the conversion of 5,16-androstadien-3β-ol from pregnenolone was poorly catalyzed (<2%) in the presence of POR. Furthermore, CYB5A stimulates and enhances the formation of androstadienol in combination with P450c17 and POR in humans (12%) and pigs (15%).

It has been previously shown that CYP17A1 catalyzes the production of 17-α-hydroxyprogesterone (17αOHP) and DHEA from pregnenolone using the 17α-hydroxylase and C17,20 lyase reactions, including the conservation of 5,16-androstadien-3β-ol in a single step via the andien-β-synthase pathway [36,37,54]. In addition, CYB5A and CYB5A reductase can modulate P450C17 enzyme activities, as CYB5A allosterically interacts with the CYP17A1-POR complex to stimulate the production of 5,16-androstadien-3β-ol [55]. In this study, CYB5A in combination with P450C17 did not stimulate the synthesis of 5,16-androstadien-3β-ol from pregnenolone when POR and CYB5 reductase were omitted from the transfection assay (Figure 5). Moreover, we could not detect DHEA conversion from pregnenolone in HEK-293 and COS-7 cells with and without CYB5A using CYP17A1. It might be due to the lack of CYP17A1-POR complex for receiving and transferring electrons, as POR transfers electrons from NADP to microsomal P450 enzymes and the only redox partner for all microsomal P450s enzymes [56]. Moreover, CYB5A accepts an electron from POR or CYB5A reductase and transfers them to CYP17A1 [57]. It is also noted that CYB5A neither stimulates nor inhibits the catalytic activity of CYP17A1 depending on the substrate and specific enzymes involved in the reaction [58]. Overall, our results showed that mouse deer CYP17A1 catalyzes the formation of 5,16-androstadien-3β-ol from pregnenolone using glucose-6-phosphate salt, glucose-6-phosphate dehydrogenase, and NADP salt cofactors.

The homology of the CYP17A1 amino acid sequences between mouse deer and pigs was 73.87%, with five extra amino acids and the deletion of two amino acids at the 21st and 22nd positions in the mouse deer as compared to the pig (Figure 3). The mouse deer’s CYP17A1 was able to transform pregnenolone to 5,16-androstadien-3β-ol with observed additions and deletions of amino acids. We have also observed that the CYP17A1 in humans (508aa) and pigs (509aa) has a single amino acid difference, but such difference would not affect the enzymatic activity for the conversion of 5,16-androstadien-3β-ol from pregnenolone [59,60]. Furthermore, to investigate the effects of additions and deletions of amino acids in mouse deer CYP17A1, we performed expression of mouse deer CYP17A1 and found that expression was upregulated and restricted to gonads (testis and ovary) compared to other tissues (heart). However, the expression was higher in the ovary than the testis (Figure 6). Robic et al. [40] showed that mRNA expression of pig CYP17A1 was restricted to the testis, ovary, and adrenal tissues and equally distributed among them.

To understand the significance of androstenone in the reproductive cycles of mouse deer, an enzyme immunoassay was specifically developed and standardized by raising the antibody against androstenone to measure the fecal androstenone using a noninvasive method. The fecal androstenone concentration followed a cyclic pattern in which delivery and postpartum estrus could be clearly distinguished from consistently elevated androstenone levels compared to baseline prior to parturition, postpartum estrus, and mating (Figure 7). Dehnhard et al., 2001 [11] identified two volatile androgen-based pheromones 5α-androst-2-en-17-one and 5α-androst-2-en-17β-ol, in the urine of female Asian elephants, which induced behavioral responses in elephant bulls. It has been demonstrated that these two urinary pheromones were involved in estrus-related activity and positively associated with urinary pregnanetriol to indicate the luteal phase and reflected the ovarian cyclicity in females (11). Based on the significantly higher concentrations of fecal androstenone during parturition and mating, this indicates that the androstenone could play a role in postpartum estrus and mating behavior in female mouse deer either alone or in coordination with estrogens. However, future studies on behavioral observation in response to androstenone in free-range animals are required to prove these findings. Moreover, all individual females showed an estrogens peak within 1.33 ± 0.18 (*n* = 12) days following parturition. However, the peak in fecal estrogens levels could be of follicular origin and presumably associated with luteinizing hormones, as mating was observed within 4–6 h of parturition. A similar observation was reported in tammar wallabies (*Macropus eugenii*), whereby mating occurred within the first 1–6 h (1.3 ± 0.8 h) of birth [61]. In addition, serum estradiol measured in tammar wallabies showed a rise in estradiol levels two days prior to parturition and peaked one to two days postpartum and declined by day three postpartum. The peak in serum estradiol levels was suggested to be of follicular origin [62]. In tammar wallabies, ovulation usually occurs between 43 and 60 h postpartum [63], and sperm reach the cervix within 40 min of copulation, the uterus in 4 h, lower oviduct in 6 h, and maintained in the uterus for over 40 h [64,65,66]. A similar scenario could be hypothesized for mouse deer that after successful copulation, spermatozoa are presumably stored for a few hours in the reproductive tract until ovulation and fertilization occur. The phenomenon of storage of sperm in a specialized structure of the reproductive tract in females is well-known and reported in many species [67].

In addition, the present study observed a positive correlation in 10 deliveries out of 13 (*n* = 6) between mean fecal androstenone and estrogens concentrations one week prior to parturition and postpartum estrus (Figure 9). This positive association could be due to the relationship of the combined synthesis of closely related sex steroids and pheromones. Babol et al., 1999 [10] reported a positive correlation between plasma androstenone and the synthesis of plasma androgens and estrogens in male pigs. Estrogens are produced from androgens by aromatization using aromatase enzyme and are involved in the regulation of the accessory sex glands of male pigs [68]. In addition, the salivary gland and fat showed a positive correlation between androstenone, androgens, and estrogens in pigs [69,70]. Similarly, adult males which were caged together with females for mating during parturition showed a positive correlation in all individuals (*n* = 3) between fecal androstenone and androgens one week prior to mating in females. As fecal estrogens and androstenone peak precisely following estrus, the decline in progesterone levels is an effective stimulant for estrus behavior and provides necessary preconditions for estrus and mating (Figure 12). Our hormone analysis also supports the previous findings on postpartum estrus in female mouse deer [41]. 

In conclusion, the current study discovered 16-androstenes in mouse deer and further sheds light on the molecular pathway of androstenone and a possible role of 16-androstenes in the reproductive cycles of mouse deer. Furthermore, mouse deer are solitary and nocturnal animals, and they can possibly use odorous 16-androstenes for sexual communication and the advertisement of their reproductive status to the opposite sex. However, further study is warranted to investigate the effects of 16-androstenes on the behavioral responses or physiological changes in free-range mouse deer. Nevertheless, our findings might help in various captive breeding programs undertaken by Indian zoos and elsewhere.

## Figures and Tables

**Table 1:** Details of animal ID, age, sex, number of fecal pellets collected and date of parturition, mating, and postpartum estrus.

S. No.	Animal ID/Name	Age (In Years)	Sex	No. of Samples Collected	Date of Parturition and Mating Observed
1	Reena	5.6	F	161	17 September 2019, 24 February 2020
2	Pinky	3.2	F	179	25 October 2019, 12 April 2020
3	Tejaswini	5.2	F	201	14 December 2019, 19 May 2020
4	Padma	5.4	F	139	24 February 2020, 23 July 2020
5	Vaneja	3.8	F	36	31 May 2020
6	Kavya	1.1	F	130	03 May 2020, 04 November 2020
7	Harshita	3.11	F	87	21 February 2020
8	Sameera	3.1	F	123	12 April 2020, 13 September 2020
9	Rinky	4.1	F	98	-
10	Prameela	5.3	F	123	-
11	Rahul	7	M	85	-
12	Shashank	5.3	M	106	-
13	Vijay	5.2	M	82	-
14	Joshi	3.2	M	77	-

**Table 2:** Primers used for the amplification of sequences of CYP17A1 and CYB5A for PCR, cloning, and quantitative PCR for relative quantification of CYP17A1 and β-actin in mouse deer.

S. No.	Primer	Forward Primer (5′ to 3′)	Reverse Primer (5′ to 3′)	Purpose
1	Mouse deer CYP17A1	ATGTGGGTGCTCGT	CATAGGGTGGAGTTTCGAGG	PCR and Cloning
2	Mouse deer CYB5A	ATGGCCGAGGAG	GTTTTCCGACGTGTAGAGGTG	PCR and Cloning
3	Pig CYP17A1	ATGTGGGTGCTCTTG	GGAGGTACTCCCCTCAGTG	PCR and Cloning
4	Pig CYB5A	ATGGCCGAACAGT	GTTTTCCGATGTGTAGAAGTG	PCR and Cloning
5	Mouse deer CYP17A1	TATCATTGACTCCAGCATTGGC	AAGCGCTCAGGCATGAACAG	qPCR and gene expression
6	β-actin	TCCTTCCTGGGCATGGAATC	GGCGCGATGATCTTGATCTTC	qPCR and gene expression

**Table 3:** Cross-reactivity of Androstenone to other C19 and various steroids.

S. No.	Steroid	Crossreactivity (%)
1	Androstenone	100.0
2	4, 16-androstadien-3-one	164.0
3	Androst-4-ene-3,17-dione	32.7
4	5α-androst-16-en-3α-ol	12.0
5	Progesterone	9.44
6	5, 16-Androstadien-3β-ol	6.47
7	Testosterone	3.33
8	5α-dihydrotestosterone	2.0
9	5-androstenediol	<1.0
10	Estradiol	<1.0

## Data Availability

The data analyzed during the current study are available from the corresponding author upon reasonable request. Further, the sequence datasets generated and analyzed during the current study are also available in the GenBank repository, https://www.ncbi.nlm.nih.gov/genbank (accessed on 18 November 2022) with accession numbers provided in this article.

## Supplementary Materials

The following supporting information can be downloaded at: https://www.mdpi.com/article/10.3390/cells11233837/s1, Figure S1. Parallel displacement curves between the pooled serial dilution of fecal ex-tracts of mouse deer (square) and respective standards (circle) of (a) Androstenone (b) Estrogens (c) Progestogens (d) Androgens, Figure S2. High-performance liquid chromatography separation of immunoreactive (a) Androstenone (b) Estrogens (c) Progestogens (d) Androgens in fecal extracts (dark line) of mouse deer and respective standards (dotted line).

## References

[1] Karlson P., Lüscher M. (1959). ‘Pheromones’: A new term for a class of biologically active substances. Nature.

[2] Kreigenhofer B.M. (2011). Exploring Social Interactions and Olfactory Communication in the Common Brushtail Possum: Implications for Management: A Thesis Presented in Partial Fulfilment of the Requirements for the Degree of Master of Science in Conservation Biology, Massey University, Albany, New Zealand. Doctoral Dissertation.

[3] van den Hurk R. (2011). Intraspecific Chemical Communication in Vertebrates with Special Attention to Sex Pheromones.

[4] Raihani G., González D., Arteaga L., Hudson R. (2009). Olfactory guidance of nipple attachment and suckling in kittens of the domestic cat: Inborn and learned responses. Dev. Psychobiol. J. Int. Soc. Dev. Psychobiol..

[5] Da Silva M.C., Canário A.V.M., Hubbard P.C., Gonçalves D.M.F. (2021). Physiology, endocrinology and chemical communication in aggressive behaviour of fishes. J. Fish Biol..

[6] Gosling L.M., Roberts S.C. (2001). Scent-marking by male mammals: Cheat proof signals to competitors and mates. Adv. Study Behav..

[7] Hu B., Mo Z., Jiang J., Liang J., Wei M., Zhu X., Liang Y., Liu Y., Huang Q., Ouyang Y. (2022). The pheromone affects reproductive physiology and behavior by regulating hormone in juvenile mice. Growth Factors.

[8] Chung-Davidson Y.W., Bussy U., Fissette S.D., Li W. (2020). Sex-dependent pheromonal effects on steroid hormone levels in sea lampreys (*Petromyzon marinus*). Gen. Comp. Endocrinol..

[9] McGlone J.J., Aviles-Rosa E.O., Archer C., Wilson M.M., Jones K.D., Matthews E.M., Gonzalez A.A., Reyes E. (2020). Understanding Sow Sexual Behavior and the Application of the Boar Pheromone to Stimulate Sow Reproduction. Animal Reproduction in Veterinary Medicine.

[10] Babol J., Squires E.J., Lundtröm K. (1999). Relationship between metabolism of androstenone and skatole in intact male pigs. J. Anim. Sci..

[11] Dehnhard M., Heistermann M., Goritz F., Hermes R., Hildebrandt T., Haber H. (2001). Demonstration of 2-unsaturated C~1~9-steroids in the urine of female Asian elephants, Elephas maximus, and their dependence on ovarian activity. Reprod.-Camb..

[12] Fadem B.H. (1987). Activation of estrus by pheromones in a marsupial: Stimulus control and endocrine factors. Biol. Reprod..

[13] Rasmussen L.E.L., Lee T.D., Zhang A., Roelofs W.L., Daves G.D. (1997). Purification, identification, concentration and bioactivity of (Z)-7-dodecen-1-yl acetate: Sex pheromone of the female Asian elephant, Elephas maximus. Chem. Senses.

[14] McGlone J.J., Devaraj S., Garcia A. (2019). A novel boar pheromone mixture induces sow estrus behaviors and reproductive success. Appl. Anim. Behav. Sci..

[15] Archunan G., Rajagopal T. (2013). Detection of estrus in Indian blackbuck: Behavioural, hormonal and urinary volatiles evaluation. Gen. Comp. Endocrinol..

[16] Kavaliers M., Kinsella D.M. (1995). Male preference for the odors of estrous female mice is reduced by the neurosteroid pregnenolone sulfate. Brain Res..

[17] Sankar R., Archunan G. (2008). Identification of putative pheromones in bovine (Bos taurus) faeces in relation to estrus detection. Anim. Reprod. Sci..

[18] Mozūraitis R., Kutra J., Borg-Karlson A.K., Būda V. (2017). Dynamics of putative sex pheromone components during heat periods in estrus-induced cows. J. Dairy Sci..

[19] Melrose D.R., Reed H.C., Patterson R.L.S. (1971). Androgen steroids associated with boar odour as an aid to the detection of oestrus in pig artificial insemination. Br. Vet. J..

[20] Perry G.C., Patterson R.L.S., MacFie H.J.H., Stinson C.G. (1980). Pig courtship behaviour: Pheromonal property of androstene steroids in male submaxillary secretion. Anim. Sci..

[21] Patterson R.L.S. (1968). 5α-androst-16-ene-3-one:—Compound responsible for taint in boar fat. J. Sci. Food Agric..

[22] Claus R. (1970). Bestimmung von Testosteron und 5a.-Androst-16-en-3-on, Einem Ebergeruchsstoff, bei Schweinen. (Estimation of testosterone and 5a.-androst-16-en-3-one, an Odourus Compound, in Pigs). Ph.D. Thesis.

[23] Reed H.C.B., Melrose D.R., Patterson R.L.S. (1974). Androgen steroids as an aid to the detection of oestrus in pig artificial insemination. Br. Vet. J..

[24] Dehnhard M., Rohrmann H., Kauffold J. (2013). Measurement of 16-Androstenes (5α-Androst-16-en-3-One, 5α-Androst-16-en-3α-ol, 5α-Androst-16-en-3β-ol) in Saliva of German Landrace and Göttingen Minipig Boars. Chemical Signals in Vertebrates 12.

[25] Nixon A., Mallet A.I., Gower D.B. (1998). Simultaneous quantification of five odorous steroids (16-androstenes) in the axillary hair of men. J. Steroid Biochem..

[26] Gower D.B., Ruparelia B.A. (1993). Olfaction in humans with special reference to odorous 16-androstenes: Their occurrence, perception and possible social, psychological and sexual impact. J. Endocrinol..

[27] Preti G., Wysocki C.J., Barnhart K.T., Sondheimer S.J., Leyden J.J. (2003). Male axillary extracts contain pheromones that affect pulsatile secretion of luteinizing hormone and mood in women recipients. Biol. Reprod..

[28] Morofushi M., Shinohara K., Funabashi T., Kimura F. (2000). Positive relationship between menstrual synchrony and ability to smell 5α-androst-16-en-3α-ol. Chem. Senses.

[29] Benton D. (1982). The influence of androstenol—A putative human pheromone—On mood throughout the menstrual cycle. Biol. Psychol..

[30] Savic I., Berglund H. (2010). Androstenol–a steroid derived odor activates the hypothalamus in women. PLoS ONE.

[31] Booth W.D. (1984). Sexual dimorphism involving steroidal pheromones and their binding protein in the submaxillary salivary gland of the Göttingen miniature pig. J. Endocrinol..

[32] Kirkwood R.N., Hughes P.E., Booth W.D. (1983). The influence of boar-related odours on puberty attainment in gilts. Anim. Sci..

[33] Beaton A.A., Jones L., Benton D., Richards G. (2022). Judgements of attractiveness of the opposite sex and nostril differences in self-rated mood: The effects of androstenol. Biol. Psychol..

[34] Robic A., Larzul C., Bonneau M. (2008). Genetic and metabolic aspects of androstenone and skatole deposition in pig adipose tissue: A review (Open Access publication). Genet. Sel. Evol..

[35] Katkov T., Gower D.B. (1970). The biosynthesis of androst-16-enes in boar testis tissue. Biochem. J..

[36] Meadus W.J., Mason J.I., Squires E.J. (1993). Cytochrome P450c17 from porcine and bovine adrenal catalyses the formation of 5, 16-androstadien-3β-ol from pregnenolone in the presence of cytochrome b5. J. Steroid Biochem. Mol. Biol..

[37] Lee-Robichaud P., Wright J.N., Akhtar M.E., Akhtar M. (1995). Modulation of the activity of human 17 α-hydroxylase-17, 20-lyase (CYP17) by cytochrome b 5: Endocrinological and mechanistic implications. Biochem. J..

[38] Nakajin S., Shively J., Yuan P.M., Hall P.F. (1981). Microsomal cytochrome P-450 from neonatal pig testis: Two enzymic activities (17. alpha. -hydroxalase and C17, 20 associated with one protein. Biochemistry.

[39] Soucy P., Luu-The V. (2002). Assessment of the ability of type 2 cytochrome b5 to modulate 17, 20-lyase activity of human P450c17. J. Steroid Biochem. Mol. Biol..

[40] Robic A., Feve K., Louveau I., Riquet J., Prunier A. (2016). Exploration of steroidogenesis-related genes in testes, ovaries, adrenals, liver and adipose tissue in pigs. Anim. Sci. J..

[41] Parvathi S., Rao M., Kumar V., Umapathy G. (2014). Observations on reproductive performance of Indian mouse deer (Moschiola indica) in captivity. Curr. Sci..

[42] Claus R., Kaufmann B., Dehnhard M., Spitzer V. (1999). Demonstration of 16-Unsaturated C-19 Steroids (‘Boar Pheromones’) in Tissues of the Male Camel (*Camelus dromedarius*). Reprod. Domest. Anim..

[43] Kusuda S., Adachi I., Fujioka K., Nakamura M., Amano-Hanzawa N., Goto N., Furuhashi S., Doi O. (2013). Reproductive characteristics of female lesser mouse deers (*Tragulus javanicus*) based on fecal progestagens and breeding records. Anim. Reprod. Sci..

[44] Weingrill T., Gray D.A., Barrett L., Henzi S.P. (2004). Fecal cortisol levels in free-ranging female chacma baboons: Relationship to dominance, reproductive state and environmental factors. Horm. Behav..

[45] McCarthy T.W., Chou H.C., Brendel V.P. (2019). SRAssembler: Selective Recursive local Assembly of homologous genomic regions. BMC Bioinform..

[46] Billen M.J., Squires E.J. (2009). The role of porcine cytochrome b5A and cytochrome b5B in the regulation of cytochrome P45017A1 activities. J. Steroid Biochem. Mol. Biol..

[47] Dawson E.C., Denissen A.E., van Weemen B.K. (1978). A simple and efficient method for raising steroid antibodies in rabbits. Steroids.

[48] Umapathy G., Kumar V., Kabra M., Shivaji S. (2013). Detection of pregnancy and fertility status in big cats using an enzyme immunoassay based on 5α-pregnan-3α-ol-20-one. Gen. Comp. Endocrinol..

[49] Kumar V., Reddy V.P., Kokkiligadda A., Shivaji S., Umapathy G. (2014). Non-invasive assessment of reproductive status and stress in captive Asian elephants in three south Indian zoos. Gen. Comp. Endocrinol..

[50] Kumar V., Buragohain S., Deka P.J., Narayan G., Umapathy G. (2021). Non-invasive reproductive hormone monitoring in the endangered pygmy hog (*Porcula salvania*). Animals.

[51] Kumar V., Sood S., Vasudevan K., Umapathy G. (2021). A practical method for storage, preservation and transportation of anuran urine samples using filter paper for hormone analysis. MethodsX.

[52] Umapathy G., Deepak V., Kumar V., Chandrasekhar M., Vasudevan K. (2015). Endocrine profiling of endangered tropical chelonians using noninvasive fecal steroid analyses. Chelonian Conserv. Biol..

[53] Abraham G.E. (1969). Solid-phase radioimmunoassay of estradiol-17β. J. Clin. Endocrinol. Metab..

[54] Nakajin S., Takahashi M., Higashiyama K., Shinoda M. (1985). Evidence for involvement of cytochrome P-450-linked oxygenase system in the conversion of C21-steroids to Δ16-C19-steroids catalyzed by pig testicular microsomes. J. Biochem..

[55] Storbeck K.H., Swart A.C., Fox C.L., Swart P. (2015). Cytochrome b5 modulates multiple reactions in steroidogenesis by diverse mechanisms. J. Steroid Biochem. Mol. Biol..

[56] Gilep A.A., Sushko T.A., Usanov S.A. (2011). At the crossroads of steroid hormone biosynthesis: The role, substrate specificity and evolutionary development of CYP17. Biochim. Biophys. Acta (BBA)-Proteins Proteom..

[57] Schenkman J.B., Jansson I. (2003). The many roles of cytochrome b5. Pharmacol. Ther..

[58] Im S.C., Waskell L. (2011). The interaction of microsomal cytochrome P450 2B4 with its redox partners, cytochrome P450 reductase and cytochrome b5. Arch. Biochem. Biophys..

[59] Conley A.J., Graham-Lorence S.E., Kagimoto M., Lorence M.C., Murry B.A., Oka K., Sanders D., Mason J.I. (1992). Nucleotide sequence of a cDNA encoding porcine testis 17 alpha-hydroxylase cytochrome P-450. Biochim. Biophys. Acta.

[60] Chung B.C., Picado-Leonard J., Haniu M., Bienkowski M.H.P.F., Hall P.F., Shively J.E., Miller W.L. (1987). Cytochrome P450c17 (steroid 17 alpha-hydroxylase/17, 20 lyase): Cloning of human adrenal and testis cDNAs indicates the same gene is expressed in both tissues. Proc. Natl. Acad. Sci. USA.

[61] Rudd C.D. (1994). Sexual behaviour of male and female tammar wallabies (*Macropus eugenii*) at post-partum oestrus. J. Zool..

[62] Shaw G., Renfree M.B. (1984). Concentrations of oestradiol-17β in plasma and corpora lutea throughout pregnancy in the tammar, *Macropus eugenii*. Reproduction.

[63] Renfree M.B., Lewis A.M. (1996). Cleavage in vivo and in vitro in the marsupial *Macropus eugenii*. Reprod. Fertil. Dev..

[64] Tyndale-Biscoe C.H., Rodger J.C. (1978). Differential transport of spermatozoa into the two sides of the genital tract of a monovular marsupial, the tammar wallaby (*Macropus eugenii*). Reproduction.

[65] Paris D.B., Taggart D.A., Shaw G., Temple-Smith P.D., Renfree M.B. (2005). Birth of pouch young after artificial insemination in the tammar wallaby (*Macropus eugenii*). Biol. Reprod..

[66] Paris D.B., Taggart D.A., Paris M.C., Temple-Smith P.D., Renfree M.B. (2005). Sperm transport, size of the seminal plug and the timing of ovulation after natural mating in the female tammar wallaby *Macropus eugenii*. Reprod. Fertil. Dev..

[67] Birkhead T.R., Møller A.P. (1993). Sexual selection and the temporal separation of reproductive events: Sperm storage data from reptiles, birds and mammals. Biol. J. Linn. Soc..

[68] Booth W.D. (1983). Development of some male characteristics supported by oestrone but not dehydroepiandrosterone in the boar. Reproduction.

[69] Bonneau M. (1982). Compounds responsible for boar taint, with special emphasis on androstenone: A review. Livest. Prod. Sci..

[70] Squires E.J., Gullett E.A., Fisher K.R.S., Partlow G.D. (1991). Comparison of androst-16-ene steroid levels determined by a colorimetric assay with boar taint estimated by a trained sensory panel. J. Anim. Sci..

