# Supplementary material for: Discovery of 16-Androstenes (Androstenone and Androstenol), Their Synthesis Pathway, and Possible Role in Reproduction of Mouse Deer (Moschiola indica)
Source: Cells. 2022 Nov 29;11(23):3837. doi: 10.3390/cells11233837 (PMC9735587; doi:10.3390/cells11233837)
Supplement: Supplementary file 1 [file cells-11-03837-s001.zip › cells-2074748-supplementary.pdf]

Supplementary Materials: All the enzyme immunoassays were validated by demonstrating parallelism between pooled standards and serial dilution of fecal extracts ($r^2 = 0.99$) (Supplementary Figure 1)

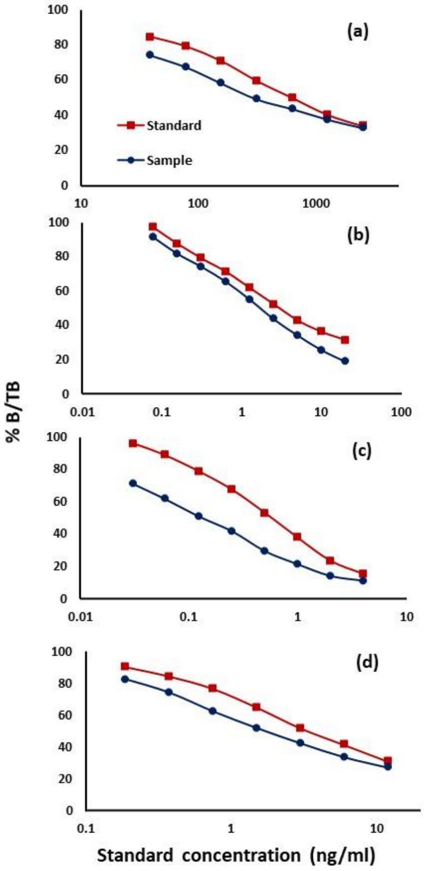

Figure S1. Parallel displacement curves between the pooled serial dilution of fecal extracts of mouse deer (square) and respective standards (circle) of (a) Androstenone (b) Estrogens (c) Progestogens (d) Androgens

High-performance liquid chromatography for EIAs validation

HPLC was performed to identification, separation, and evaluation of immunoreactivity of fecal androstenone, progesterone, estradiol, and testosterone with the corresponding antibodies using the Shimadzu CTO-10AS system (Shimadzu Corporation, Tokyo, Japan). Fecal hormones were separated and identified using steroid-specific reverse-phase C-18 column (waters column, symmetry C-18, 4.6 x 20 mm, 3.5 mm, intelligent speed (IS) column. Prior to HPLC, pooled fecal samples were purified and passed through Sep-Pak C18 cartridges (Waters, Milford, MA, USA) and eluted with absolute methanol as described previously (45). The purified supernatant was dried in nitrogen gas, reconstituted in 100µl of

absolute methanol and vortexed for 1 min. Respective standards and fecal samples were injected into the HPLC and eluted using a gradient flow of 20–94% acetonitrile (ACN): water (H2O) for 8 min at a flow rate of 1 mL/min. Hormones were detected at the 190 to 400 nm wavelength and eluted fractions of 250µl were collected every 15 seconds (4 fractions/minute) and vacuum dried. The dried samples were resuspended in 100 µL of EIA buffer to evaluate the immunoreactivity using the corresponding hormone EIA (Figure S2).

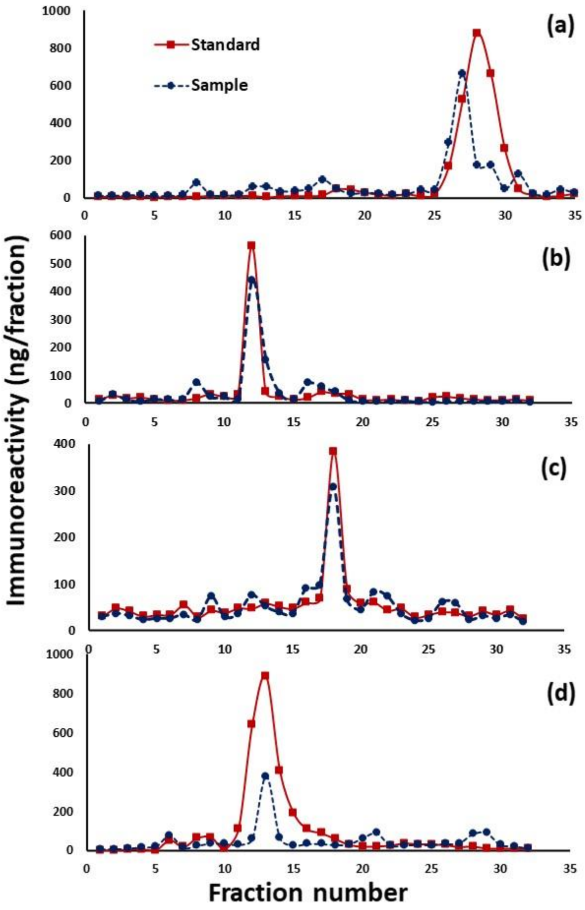

Figure S2. High-performance liquid chromatography separation of immunoreactive (a) Androstenone (b) Estrogens (c) Progestogens (d) Androgens in fecal extracts (dark line) of mouse deer and respective standards (dotted line).
